# Mononuclear phagocyte regulation by the transcription factor Blimp‐1 in health and disease

**DOI:** 10.1111/imm.13249

**Published:** 2020-09-27

**Authors:** Isabel Ulmert, Luís Henriques‐Oliveira, Carlos‐Filipe Pereira, Katharina Lahl

**Affiliations:** ^1^ Division of Biopharma Institute for Health Technology Technical University of Denmark (DTU) Kongens Lyngby Denmark; ^2^ Center for Neuroscience and Cell Biology University of Coimbra Coimbra Portugal; ^3^ Cell Reprogramming in Hematopoiesis and Immunity Laboratory Lund Stem Cell Center, Molecular Medicine and Gene Therapy Lund University Lund Sweden; ^4^ Wallenberg Center for Molecular Medicine Lund University Lund Sweden; ^5^ Immunology Section Lund University Lund Sweden

**Keywords:** Blimp‐1, dendritic cells, immune regulation, macrophages, transcriptional regulation

## Abstract

B lymphocyte‐induced maturation protein‐1 (Blimp‐1), the transcription factor encoded by the gene *Prdm1*, plays a number of crucial roles in the adaptive immune system, which result in the maintenance of key effector functions of B‐ and T‐cells. Emerging clinical data, as well as mechanistic evidence from mouse studies, have additionally identified critical functions of Blimp‐1 in the maintenance of immune homeostasis by the mononuclear phagocyte (MNP) system. Blimp‐1 regulation of gene expression affects various aspects of MNP biology, including developmental programmes such as fate decisions of monocytes entering peripheral tissue, and functional programmes such as activation, antigen presentation and secretion of soluble inflammatory mediators. The highly tissue‐, subset‐ and state‐specific regulation of Blimp‐1 expression in MNPs suggests that Blimp‐1 is a dynamic regulator of immune activation, integrating environmental cues to fine‐tune the function of innate cells. In this review, we will discuss the current knowledge regarding Blimp‐1 regulation and function in macrophages and dendritic cells.

## Graphical abstract

Blimp‐1 shows a broad expression pattern in mononuclear phagocytes (MNPs), and is emerging as an important rheostat for innate immune cell subset identity, activation and function, in addition to its known roles in the adaptive immune system. We here discuss the emerging picture of Blimp‐1 in integrating environmental triggers and imprinting context‐specific function of MNPs for the maintenance of immune homeostasis.

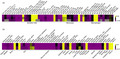

**Abbreviations**
AhR: aryl hydrocarbon receptor
ATAC‐seq: assay for transposase‐accessible chromatin sequencing
Bcl‐6: B‐cell lymphoma 6
Blimp‐1: B lymphocyte‐induced maturation protein‐1
BMDC: bone marrow‐derived dendritic cell
BMP: bone morphogenic protein
cDC: conventional dendritic cell
ChIP: chromatin immunoprecipitation
CIITA: Class II major histocompatibility complex transactivator
CST3: cystatin C
CTSS: cathepsin S
ERα: estrogen receptor alpha
GM‐CSF: granulocyte‐macrophage colony‐stimulating factor
GWAS: genome‐wide association studies
Hobit: homologue of Blimp‐1 in T‐cells
IBD: inflammatory bowel disease
IRF4: interferon regulatory factor 4
IRF8: interferon regulatory factor 8
LN: lymph node
M‐CSF: macrophage colony‐stimulating factor
miRNA: microRNA
MNP: mononuclear phagocyte
Mo‐DC: monocyte‐derived dendritic cell
Mo‐Mac: monocyte‐derived macrophage
NK cells: natural killer cells
NLR: NOD‐like receptor
NLRP12: NLR family pyrin domain containing 12
PRDI‐BF1: positive regulatory domain 1‐binding factor 1
Prdm1: positive regulatory domain containing 1, with zinc‐finger domain
PRR: pattern recognition receptor
RANK: receptor activator of nuclear factor kappa‐Β
RANKL: receptor activator of nuclear factor kappa‐Βnonbreakingspaceligand
SLE: systemic lupus erythematosus
SNP: single nucleotide polymorphism
SOCS1: suppressor of cytokine signalling 1
TLR: Toll‐like receptor
Tregs: regulatory T‐cells

## Introduction

B‐lymphocyte‐induced maturation protein‐1 (Blimp‐1) was first described in 1991 as a potent virus‐induced interferon β (IFNβ) repressor in humans. The 88‐kD protein containing five zinc‐finger motifs was designated PRDI‐BF1 (positive regulatory domain 1‐binding factor 1), due to its specific binding to the PRDI element in the IFNβ promotor.^1^ Shortly thereafter, Mark Davis and colleagues identified a transcriptional repressor in the mouse expressed in late‐stage mature B‐cells and plasma cells, and named it Blimp‐1.^2^ The mouse and human versions of Blimp‐1, encoded by the gene *Prdm1* (positive regulatory domain containing 1, with zinc‐finger domain), are highly homologous.^3^ Blimp‐1 serves as a transcriptional and epigenetic regulator of target genes across multiple cell types. It can directly bind DNA and recruit chromatin‐modifying factors associated with inhibition of gene transcription, including histone deacetylases 1 and 2 (HDAC1/2), G9a histone methyltransferase and Groucho family proteins.^4^, ^5^, ^6^ In this review, we will discuss the role of Blimp‐1 in regulating mononuclear phagocyte (MNP) development and function in health and disease. Unless otherwise stated, we focus this review on knowledge derived from murine experiments.

## Blimp‐1 is broadly expressed, and fulfills many different roles across various cell types

Blimp‐1 is expressed across many haematopoietic and non‐haematopoietic cell types, and fulfills a broad array of functions. A growing body of literature covers the role of Blimp‐1 as an important regulator during early developmental processes, across vertebrate species (reviewed in detail in Ref. [7]). Murine embryos deficient for Blimp‐1 die at about embryonic day 10·5 due to placental insufficiency.^8^, ^9^ Dose‐dependent bone morphogenetic protein (BMP)/Smad‐induced Blimp‐1 expression is essential for primordial germ cell specification,^8^, ^10^ where it acts in concert with the transcription factors AP2γ and PRDM14·^11^ Blimp‐1 is also broadly expressed in multipotent progenitor cells during tissue development, and guides morphogenesis of various tissues, including the posterior forelimb, the caudal pharyngeal arches, the cardiac outflow tract and the sensory vibrissae.^12^

Blimp‐1 specifically plays important roles in epithelial cell differentiation and polarization. During the suckling phase, Blimp‐1 is essential in maintaining the neonatal phenotype of intestinal epithelial cells. Epithelial cell‐specific Blimp‐1 deficiency leads to neonatal growth retardation and mortality owing to dysregulated expression of genes associated with metabolic functions.^13^, ^14^ Blimp‐1 also represses expression of MHC Class I pathway genes, by directly competing with interferon regulatory factor (IRF)1 in the neonatal intestinal epithelium, thereby contributing to neonatal immune tolerance.^15^ Outside of the intestine, Blimp‐1 is also important for mammary gland formation by supporting proliferation and polarization of rare luminal progenitors.^16^ Based on experiments using *in vitro* organoids, this is in part due to elevated IFNλ expression in the epithelial cells.^17^ In the cancerous mammary epithelium‐derived cell line MCF7, high RelB/NFκB levels induce Blimp‐1 expression, which in turn suppresses the estrogen receptor α (ERα), driving elevated migratory capacity due to reduced levels of E‐cadherin and γ‐catenin.^18^ Transforming growth factor (TGF)β‐induced epithelial‐to‐mesenchymal transition in breast cancer cells is also orchestrated by Blimp‐1: here, Blimp‐1 represses BMP‐5, leading to deregulation of Snail.^19^ In the homeostatic skin, Blimp‐1 has been shown to regulate sebaceous gland homeostasis by directly repressing c‐Myc in sebocyte progenitors,^20^ and it regulates the final steps of cornification, allowing for terminal epidermal differentiation.^21^ Thus, Blimp‐1 influences steady‐state and pathogenic epithelial cell development and function at multiple levels. This heterogeneous functionality in developmentally related cell types, as depicted here across epithelial cells, suggests a highly contextual action of Blimp‐1.

Despite its broad expression and diverse functional impacts within the non‐haematopoietic system, Blimp‐1 is still best known for its crucial role as a key regulator of plasma cell development. During the differentiation of B‐cells into plasma cells, IRF4 directly induces Blimp‐1 expression,^22^ and IRF4 and Blimp‐1 are together required for the induction and maintenance of functional plasma cells.^23^, ^24^ Blimp‐1 represses B‐cell lymphoma 6 (Bcl‐6) and c‐Myc, key factors supporting germinal centre reactions, thereby allowing for the terminal differentiation of the plasma cell.^25^, ^26^ Importantly, Bcl‐6 can also directly repress Blimp‐1, placing these two transcription factors into the centre of mature B‐cell trajectory decisions, together with the Blimp‐1‐inducing IRF4 and the Blimp‐1‐repressing IRF8 as upstream regulators.^27^, ^28^ A series of elegant studies showed that Blimp‐1 directly regulates numerous pathways to affect plasma cell fate and function. One key effect is an increase in the plasma cell’s capacity to produce and secrete vast amounts of antibody (reviewed in Ref. [29]). This is facilitated by Blimp‐1‐mediated upregulation of *Ire1*, which activates Xbp‐1 through splicing, driving the required unfolded protein response pathway.^30^ Other aspects of plasma cell biology regulated by Blimp‐1 include chemokine receptors and adhesion molecules: Blimp‐1 inhibits the expression of *Cxcr5*, *Ccr7*, *S1pr1*, *Sd22*, *Itgb7* and *Sell*, strongly suggesting that it affects the positioning of plasma cells after their maturation in secondary lymphoid organs.^29^

Parallel to the expression pattern in B‐cells, Blimp‐1 also marks terminal effector T‐cells, although with the exception of T follicular helper cells, which require high expression of the mutually exclusive transcription factor Bcl‐6 (reviewed in Refs [31, 32]). A subset of regulatory T‐cells (Tregs) primarily found in mucosal tissues depends on Blimp‐1 for its high expression of interleukin (IL)‐10. Indeed, deficiency of Blimp‐1 in the T‐cell compartment leads to spontaneous colitis onset at the age of 6 weeks.^33^, ^34^ Mirroring the regulatory network in plasma cells, Blimp‐1 expression in Tregs requires induction by IRF4·^34^ In intestinal RORγt^+^ Tregs, however, Blimp‐1 has been shown to also directly inhibit IRF4 binding to the IL‐17 locus, facilitating the maintenance of the regulatory state.^35^ Likewise, Blimp‐1 can stabilize the suppressive phenotype and correct localization of follicular Tregs, allowing them to inhibit germinal centre reactions.^36^ However, the role of Blimp‐1 in follicular Tregs may be context‐dependent, as Blimp‐1 expression induces an ST2^+^ (Il1rl1, IL‐33R) allergy‐promoting phenotype of Tregs in the house dust mite model of allergic asthma.^37^ Interestingly, Blimp‐1 shows particularly high expression levels in visceral adipose tissue Tregs in male mice, where it is induced in response to, and is essential to counteract, the low‐grade inflammatory signals sent by male visceral stroma. In this context, Blimp‐1 directly induces expression of the regulatory cytokine IL‐10, the chemokine receptor CCR2 (essential for positioning the cells within CCL2‐abundant fat), and ST2 (important for the expansion of the visceral fat Treg population).^38^

In CD8 effector T‐cells, Blimp‐1 supports terminal differentiation along with high expression of effector molecules such as granzyme B.^39^, ^40^ Interestingly, IL‐2‐induced cytotoxicity in tumour‐specific cytotoxic CD4 T‐cells equally depends on Blimp‐1 expression for optimal granzyme B expression, suggesting that Blimp‐1 is generally required for the cytotoxic programme in T‐cells.^41^ Upon chronic viral infection, Blimp‐1 drives CD8 T‐cell exhaustion by directly repressing expression of the IL2rα chain and CD27·^42^, ^43^ Together with the transcription factor Hobit (homologue of Blimp‐1 in T‐cells), Blimp‐1 was shown to support the formation of tissue‐resident memory cells while suppressing circulating memory cells.^44^

Besides its profound role within the adaptive immune system, Blimp‐1 is emerging as an important rheostat for innate immune cell subset identity, activation and function. In contrast to T‐ and B‐cells, natural killer (NK) cells constitutively express Blimp‐1. Similarly to its role in T‐cells, Blimp‐1 expression in NK cells is required for high granzyme B expression, but not for the secretion of cytokines or for their lytic capability. In sharp contrast to its regulation in adaptive immune cells, Blimp‐1 expression in NK cells is independent of IRF4 and Bcl‐6. Instead, steady‐state expression of Blimp‐1 in NK cells depends on T‐bet expression, suggesting that Blimp‐1 regulation is context‐dependent across lymphocyte populations.^45^ Blimp‐1 was also shown to control the function of human NK cells, where it reportedly has broader effects: Blimp‐1 inhibits secretion of pro‐inflammatory cytokines such as tumour necrosis factor (TNF)α and IFNγ, mirroring its function in CD4 T‐cells.

Blimp‐1‐mediated polarization and regulation of terminal effector function appears to be a common modality across numerous cell lineages. In addition to the above‐mentioned lineages, genome‐wide association studies (GWAS), paired with mechanistic studies using animal models, paint an emerging picture of a role for Blimp‐1 in the regulation of antigen‐presenting cells with implications for immune homeostasis. In the remainder of this review, we will discuss the emerging role of Blimp‐1 in MNPs. These include tissue‐resident macrophages, monocyte‐derived macrophages (Mo‐Macs) and dendritic cells (Mo‐DCs), and type 1 and type 2 subsets of conventional dendritic cells (cDC1 and cDC2; nomenclature defined in Ref. [46]). cDC1 depend on IRF8 and BATF3, are characterized by their expression of XCR1, and excel at cross‐presenting antigen to CD8 T‐cells, endowing them with a unique function in orchestrating the immune response towards viruses and intracellular bacteria. cDC2 on the other hand express IRF4, are characterized by their expression of CD11b and Sirp‐α, and present antigen to CD4 T‐cells with high efficacy, leading to strong immunity particularly towards extracellular bacteria (summarized in Ref. [47]).

## Blimp‐1 in mononuclear phagocytic development

**Figure 1:**
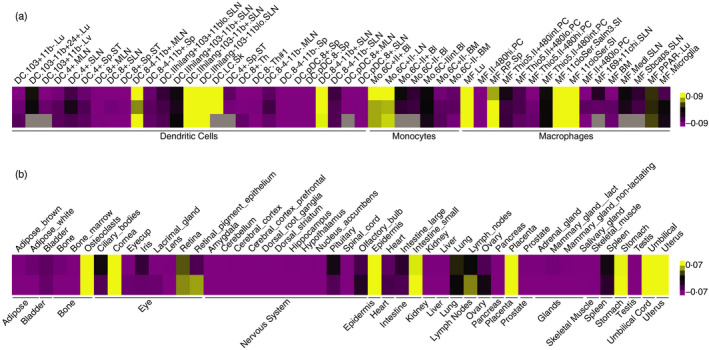
B lymphocyte‐induced maturation protein‐1 (Blimp‐1) expression across mononuclear phagocytes (MNPs) and murine tissues. (a) Heatmap showing Blimp‐1 expression in MNP populations. Dendritic cell (DC), monocyte (Mo) and macrophage (MF) samples were extracted from the Immunological Genome Project microarray dataset (ImmGen, GSE15907). MLN, mesenteric lymph node; Lu, lung; Lv, liver; SLN, skin draining lymph node; Sp, spleen; LC, Langerhans cell; Sk, skin; Th, thymus; Bl, blood; BM, bone marrow; PC, peritoneal cavity; SI, small intestine; Medl, medullary; Sbcaps, subcapsular sinus; CNS, central nervous system; Ser, serosal; Salm, Salmonella‐infected. (b) Heatmap showing Blimp‐1 expression across multiple mouse tissues (GeneAtlas, MOE430), grouped according to their origin. Yellow indicates increased expression, and purple indicates decreased expression over the mean. Data from two−three replicates are shown. Unavailable data are depicted in grey. Gene expression data analysed in Gene Cluster 3·0 and displayed with Java Treeview.

Paralleling its widespread expression in lymphocyte subsets, Blimp‐1 shows a broad expression pattern and functionality in MNPs (Fig. 1), which to date remains relatively unexplored in its complexity. An early study identified Blimp‐1 as a myeloid lineage determinant *in vitro*. Blimp‐1 expression is induced upon differentiation of pro‐myelocytic cells into either macrophages or granulocytes.^48^ Accordingly, *Prdm1* transcripts were also found to be expressed in human peripheral blood monocytes and granulocytes. Overexpression of Blimp‐1 in pro‐monocytic cells triggered the development of a partial macrophage morphology, including cell surface expression of CD11c and CD11b.^48^

Upon extravasation from the bloodstream, monocytes can differentiate into Mo‐Macs or Mo‐DCs.^46^ Interestingly, Blimp‐1 was recently discussed to act as an important positive regulator of Mo‐DC differentiation.^49^ The study showed that human monocytes express a Mo‐Mac‐biased transcriptomic signature, including MafB, CD163 and MerTK, and are by default directed towards a Mo‐Mac fate. However, microenvironmental cues such as IL‐4, TNFα and aryl hydrocarbon receptor (AhR) signalling supported a switch to an IRF4‐dependent Mo‐DC fate, a process suppressed by silencing of *Prdm1*·^49^ Although the exact signalling network underlying Blimp‐1‐induced differentiation into Mo‐DCs was not studied, the correlation between expression of IRF4 and Blimp‐1 in Mo‐DCs is unlikely to be coincidental, as IRF4 has been shown to act as a potent transcriptional activator of *Prdm1* in other settings.^22^, ^34^, ^35^, ^50^ Similarly to the pathway of B‐cell maturation,^51^ AhR signalling induced rapid *Prdm1* expression, and AhR signalling is required for the MHCII^+^ CD226^+^ subset of peritoneal MNP differentiation *in vivo*.^49^ These cells are sensitive to antimicrobial treatment and therefore assumed to depend on the microbiome. To what extent IRF4, Blimp‐1 and AhR signalling converge in supporting MNP fate decisions, and which transcriptional hierarchies define this network, remain to be investigated. In addition, given the assumption that the Blimp‐1‐sensitive subset requires microbial signalling for differentiation and is therefore likely to be influenced by environmental changes, specific analysis of Blimp‐1‐influenced monocyte fate differentiation and the specific role of Blimp‐1 in MNP maturation in other tissues than the peritoneal cavity is warranted. Importantly, Blimp‐1 has been described as a marker for a specific macrophage population (defined as CD11c^+^ CD206^int^ CD121b^+^) located near the microbe‐exposed surface in the colon,^52^ suggesting that Blimp‐1 expression predestines cells for certain fates, but does not lock MNPs into a DC phenotype per se. Instead, the accumulating evidence suggests that environmental factors, such as microbiota‐derived AhR ligands, engage a transcriptional network involving Blimp‐1 to allow for functional fine‐tuning of plastic lineages.

Whether Blimp‐1 plays a significant role in the development and subset differentiation of cDCs is unclear. A recent study found no effect on DC differentiation *in vitro* (GM‐CSF + IL‐4) upon silencing of *Prdm1*.^53^ However, the deletion of Blimp‐1 in the entire haematopoietic system resulted in the selective expansion of the cDC2 subset in spleen and peripheral lymph nodes (LNs), owing to an increased number of precursors. This may suggest that Blimp‐1 negatively regulates cDC2, but secondary effects caused by the absence of Blimp‐1 in other haematopoietic cells were not excluded.^54^ Using reporter mice for Blimp‐1, we detected high expression specifically in small intestinal cDC2, with no detectable reporter signal in splenic cDCs.^55^ Importantly, and seemingly contradictory to what was suggested by Chan *et al*.^54^, *CD11c*.cre‐driven deletion of Blimp‐1 caused a specific loss of CD103^+^ CD11b^+^ cDC2 in the small intestinal lamina propria and the corresponding migratory population in the mesenteric LNs.^55^ cDC2 are largely found in the marginal zone/bridging channel of the spleen and in the subcapsular sinus of peripheral LNs (reviewed in Ref. [56]), which are sites of relatively high antigen exposure, suggesting that steady‐state Blimp‐1 expression is a consequence of microenvironmental immune signalling. Antigen exposure in the small intestine is significantly higher, and our data suggest that high Blimp‐1 expression in cDC2 stabilizes rather than regulates the cDC2 population. Further research is required to explore whether immune activation regulates Blimp‐1 expression in cDC2 systemically, and whether this affects cDC2 abundance. Intriguingly, a recent study reported conserved expression of Blimp‐1 between mouse and man in a subset of splenic cDC2 that also expressed RORγt, again suggesting an intricate network of transcriptional regulation in cDC2 likely affected by the environment and driving the suggested heterogeneity within cDC2.^57^ While the mechanisms underlying the loss of intestinal cDC2 in the absence of Blimp‐1 are entirely unexplored, Blimp‐1 is exclusively expressed in IRF4‐dependent cDC2, suggesting that the mutual antagonism of IRF4 and IRF8 described for B‐cells and DCs alike may also result in overlapping regulatory circuits governing Blimp‐1 expression in DC subsets. Of note, expression patterns of IRF4 and Blimp‐1 are conserved across murine and human intestinal cDC2·^55^

Blimp‐1 also specifically supports the generation of osteoclasts, which are multi‐nucleated cells derived from the monocyte‐macrophage lineage responsible for bone resorption. Osteoclasts develop from the fusion of haematopoietic myeloid precursors, and differentiate in response to receptor activator of nuclear factor kappa‐B ligand (RANKL) and granulocyte‐macrophage colony‐stimulating factor (GM‐CSF). Briefly, the interaction of RANKL with receptor activator of nuclear factor kappa‐B (RANK) activates the initial expression of the master regulator NFATc1. This in turn induces expression of a gene signature essential for osteoclast differentiation and function (reviewed in Ref. [58]). The signature depends on RANK‐induced Blimp‐1 to inhibit the anti‐osteoclastogenic genes Bcl‐6, IRF8 and MafB.^59^, ^60^, ^61^ Blimp‐1 deficiency in osteoclast progenitors consequently results in dysregulation of osteoclastogenesis, evident by aberrant bone formation *in vivo*. Intriguingly, IL‐33 signalling through ST2 inhibits RANKL‐induced osteoclast differentiation of macrophage colony‐stimulating factor (M‐CSF)‐ and RANKL‐cultured bone marrow (BM)‐cells by downregulating Blimp‐1 mRNA while upregulating IRF8 expression.^62^ Although no direct signalling pathway has been proposed, this suggests Blimp‐1 functions downstream of ST2, which is in sharp contrast to cells in the T‐cell lineage mentioned above.^37^, ^38^

## Regulation of Blimp‐1 expression in mononuclear phagocytes

The molecular players driving Blimp‐1 expression specifically in the MNP compartment have not been assessed in detail. However, some data suggest that Blimp‐1 expression correlates with immune activation through pattern recognition receptors (PRRs): Toll‐like receptors (TLRs) and NOD‐like receptors (NLRs) engagement on MNPs induce Blimp‐1 in various settings. GM‐CSF‐cultured bone marrow‐derived dendritic cells (BMDCs) induced Blimp‐1 expression upon LPS, CpG, poly(I:C) and TNFα stimulation. Pharmacological inhibition of p38, MAPK and NFκB abrogated Blimp‐1 transcription in response to LPS.^54^ Similarly, M‐CSF‐cultured BM‐macrophages upregulated Blimp‐1 transcripts rapidly upon exposure to LPS or pathogens such as *Listeria monocytogenes*, *Escherichia coli*, *Staphylococcus aureus* and Sendai virus.^63^, ^64^ Interestingly, Blimp‐1 was often induced in two waves, one transient induction at 2 hr post‐infection and a second peak induction after 24 hr, suggesting that Blimp‐1 expression can result from both an immediate as well as a downstream PRR trigger.^64^ TLR2‐deficient BM‐derived macrophages pretreated with an IL‐1R antagonist failed to induce Blimp‐1 transcription upon *L. monocytogenes* infection. Likewise, inhibition of the downstream signal transducers MyD88, MAPK and NFκB fully abrogated expression of Blimp‐1. This implies that cell surface TLR2 and cytosolic PRRs regulating IL‐1β production cooperate in the control of Blimp‐1 transcription in BM‐derived macrophages upon *L. monocytogenes* infection.^64^ Context‐dependent expression of Blimp‐1 in cDC2 *in vivo* in the small intestine^55^ and inducible expression in lung cDC2 upon infection^65^ further supports the relevance of immune activation in Blimp‐1 induction.

**Figure 2:**
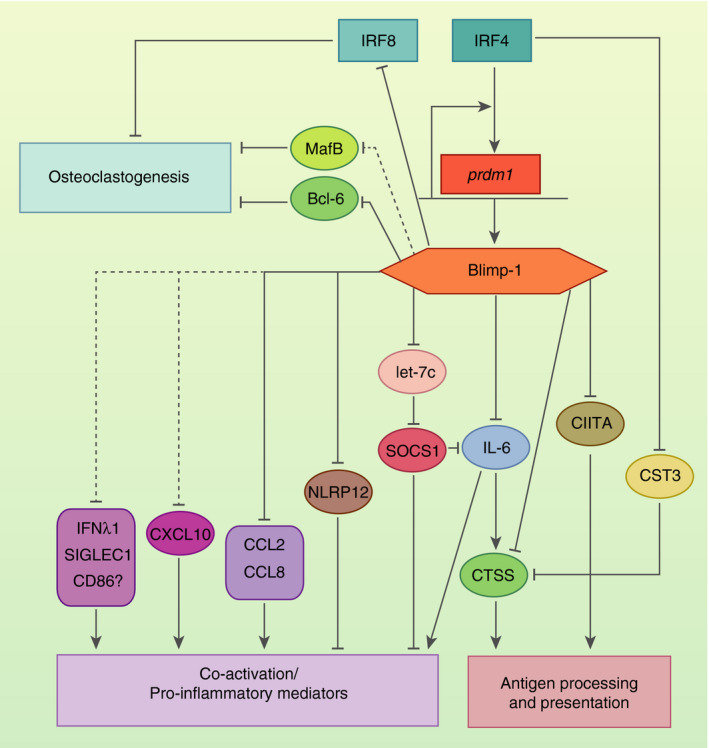
Identified B lymphocyte‐induced maturation protein‐1 (Blimp‐1) targets in mononuclear phagocytes (MNPs). Blimp‐1, encoded by *Prdm1*, represses interleukin (IL)‐6 directly and indirectly by regulating the levels of the microRNA (miRNA) let‐7c and SOCS1·^54^, ^72^ It also represses cathepsin S (CTSS) directly and indirectly via repression of IL‐6·^78^, ^79^ By downregulating CTSS and CIITA,^84^ Blimp‐1 directly influences both antigen‐processing and ‐presentation by interfering with transcription of MHC Class II‐dependent genes. Interferon regulatory factor (IRF)4 induces Blimp‐1 transcription and negatively regulates CST3,^50^ an inhibitor of CTSS, thereby allowing Blimp‐1‐mediated fine‐tuning of the antigen‐processing and ‐presentation machinery. Blimp‐1 also directly represses CCL2 and CCL8,^54^, ^64^ and has been implicated to regulate CXCL10 as well as SIGLEC1, IFNλ1 and possibly CD86,^74^ which are all components of a pro‐inflammatory response. On the other hand, Blimp‐1 can enhance activation by repressing the NFkB/TNFR pathway repressor NLRP12·^73^ These regulatory mechanisms of Blimp‐1 in the antigen‐processing and presentation as well co‐activation/pro‐inflammatory mediation, are likely to be operative upon pattern recognition receptor (PRR) engagement in MNPs. Additionally, RANK‐RANKL interaction (not shown) induces Blimp‐1‐mediated repression of anti‐osteoclastogenic genes Bcl‐6, IRF8 and MafB,^59^, ^60^, ^61^ assigning a central role to Blimp‐1 in osteoclast development. Solid lines indicate genes shown to be directly repressed by Blimp‐1, and dotted lines indicate Blimp‐1 repression by a currently unknown mechanism.

It is intriguing to postulate that some other known activators of Blimp‐1 in B‐ and T‐cells also increase its expression in the MNP compartment. As described above, this has been shown for the AhR ligand FICZ rapidly increasing *Prdm1* expression in IRF4‐dependent human monocyte cultures,^49^ and in GM‐CSF + IL‐4 monocyte cultures, where IRF4 positively regulates Blimp‐1 expression.^50^ A recent study reports activation of *Prdm1* transcription by IL‐10‐induced STAT3‐signalling in T‐cells, resulting in a T_H_2 response upon nasal triggering in the lung, but not systemically.^66^ Blimp‐1 induction might reflect one mechanism by which STAT3 regulates MNP activation, given that both STAT3^67^ and Blimp‐1 (reviewed below, Fig. 2) negatively regulate CD11c^+^ MNP function.

## Targets of Blimp‐1 in mononuclear phagocytes

### Blimp‐1 in the regulation of inflammatory mediators

Conditional deletion of Blimp‐1 in MNP subsets revealed a role for Blimp‐1 in immune homeostasis and regulation of inflammation. The identification of direct targets of Blimp‐1 repression has just begun to explain this functional importance of Blimp‐1 activity.

In M‐CSF‐cultured BM‐macrophages, chromatin immunoprecipitation (ChIP) analysis identified *Ccl8* to be a direct target of Blimp‐1, which was also evident *in vivo* as a steady‐state increase of *Ccl8* transcripts and CCL8 protein in macrophages and in sera of *Prdm1* ^fl/fl^ *LysM*.cre mice.^64^ Blimp‐1 deficiency in the myeloid lineage rendered mice less susceptible to *L. monocytogenes* infection, as elevated CCL8 attracted more IL‐17F‐producing γ/*δ* T‐cells, which in turn increased neutrophil granulopoiesis and recruitment.^64^

In GM‐CSF‐cultured BMDCs, a heterogeneous population of macrophages and DCs,^68^ Blimp‐1 directly represses *Il‐6* and *Ccl2*.^54^ Dysregulation of IL‐6 expression by DCs *in vivo* was also described in the context of inflammatory bowel disease (IBD) and systemic lupus erythematosus (SLE) studies, where CD11c^+^ cell‐specific Blimp‐1 deficiency led to enhanced immunopathology. In dextran sulphate sodium‐induced colitis, severe disease state was specifically attributed to dysregulated IL‐1β and IL‐6 secretion by colonic CD103^+^ DCs. Elevated pro‐inflammatory cytokines resulted in the enhanced influx of neutrophils and activated macrophages into the colonic tissue. These macrophages expressed higher levels of matrix metalloproteinases, as a direct consequence of increased IL‐1β and IL‐6 from Blimp‐1‐deficient colonic CD103^+^ cDCs, leading to higher tissue destruction and exacerbated inflammation.^69^ Blimp‐1‐deficient female mice spontaneously presented with an SLE‐like phenotype, which could be rescued by additional knockout of IL‐6, again suggesting the direct involvement of IL‐6 from CD11c^+^ cells.^70^ In Flt3L‐cultured BMDCs and splenic Blimp‐1‐deficient cDCs, IL‐6 production was predominantly increased in female‐derived cells upon LPS stimulation. This increased IL‐6 production was responsible for driving the expansion of Tfh cells, resulting in the increased germinal centre formation, and ultimately in higher titres of IgG(2b) autoantibodies. The gender bias in the observed autoimmune phenotype could be explained by the role of ERα signalling in the positive regulation of IL‐6 in BMDCs.^71^ Blimp‐1 also directly represses transcription of the microRNA (miRNA) let‐7c in DCs.^72^ One important let‐7c target, suppressor of cytokine signalling 1 (SOCS1), is a regulator of several cytokines acting via the JAK/STAT3 pathway. Induction of SOCS1 was abrogated in LPS‐stimulated Blimp‐1‐deficient splenic DCs as well as in BMDCs, resulting in high levels of IL‐6, TNFα and IFNγ secretion. The increased IL‐6 expression, as well as decreased SOCS1, was reversed by lentiviral reconstitution of Blimp‐1 in BMDCs placing Blimp‐1 in a let‐7c‐SOCS1‐regulated cytokine response in DCs.^72^ Together, these data suggest that Blimp‐1 can suppress IL‐6 both directly and indirectly (Fig. 2).

In contrast to intestinal cDC2, lung DCs express little Blimp‐1 expression at steady‐state (Fig. 1a), possibly reflecting a lower basal activation level of lung immune cells. As such, stimulation of the lung environment with bacterial or viral triggers drives expression of Blimp‐1 in lung cDC2.^65^ Expression of the transcription factor correlates with a ‘paralysed’ state in these cells, as measured by lower cytokine production and a reduced ability to induce CD4 T‐cell proliferation. Although the molecular targets of Blimp‐1 were not identified in this study, cDC2 regulation by Blimp‐1 likely contributes to the sepsis‐induced immunosuppression observed in the lung upon pneumonia. Importantly, Blimp‐1 expression levels in circulating cDC2 also positively correlated with the severity of secondary infection in patients.^65^

In contrast to the suggested role for Blimp‐1 in negative immune regulation, Blimp‐1 was also implicated in regulating immune suppression by the NLR ‐ NLRP12 (NLR family pyrin domain containing 12).^73^ The PRR‐mediated increase in Blimp‐1 expression leads to direct silencing of NLRP12 expression, enabling full activation through the NFκB and TNFR pathways. These findings suggest that Blimp‐1 can, in the given context, remove the break from inflammatory signalling in addition to suppressing inflammation (Fig. 2). The genetic targets of Blimp‐1 specifically in DCs are unknown. In‐depth phenotyping of remaining intestinal cDC2 in mice lacking Blimp‐1 in DCs coupled with high‐throughput single‐cell gene expression and chromatin landscape assessment will reveal whether decreased abundance of cDC2 in the absence of Blimp‐1 is due to activation or suppression of cDC activity, or whether Blimp‐1 plays a role in DC ontogeny.

Interestingly, combined RNA‐ and ATAC‐seq (assay for transposase‐accessible chromatin sequencing) analyses identified Blimp‐1 as a positive, rather than negative, upstream modulator of the IFN response during HIV infection of human GM‐CSF + IL‐4‐cultured Mo‐DCs.^74^ ShRNA‐driven inhibition of *Prdm1* resulted in defective expression of CD86 and SIGLEC1, as well as IFNL1 and CXCL10,^74^ contrasting previous *in vitro* findings by Xiao *et al*.^53^ The original finding that Blimp‐1 potently represses IFNβ in cell lines by recruiting the G9α histone methyltransferases to the IFNβ promoter,^5^ together with these novel findings, suggests that Blimp‐1‐mediated positive versus negative regulation of gene expression may be highly contextual.

### Blimp‐1 in antigen processing and presentation

In addition to regulation through cytokine production and costimulatory molecule expression, Blimp‐1 directly interferes with antigen presentation, by influencing both antigen processing and presentation by MHC Class II. The functional importance of Blimp‐1 in the regulation of antigen presentation by cDCs has received considerable attention due to its consequences for MHC‐dependent systemic autoimmunity. One of the most important molecules involved in antigen presentation is cathepsin S (CTSS), which cleaves the invariant chain to permit loading into MHC Class II molecules,^75^ and generates a pool of peptides available for presentation on MHC Class II.^76^, ^77^ In‐depth analysis of putative causes of SLE induction in female mice harbouring Blimp‐1‐deficient DCs revealed heightened expression levels of CTSS in addition to IL‐6.^70^, ^78^ Blimp‐1 represses *Ctss* in cDCs directly and indirectly via the downregulation of the IL‐6‐STAT3 signalling pathway (Fig. 2).^78^, ^79^ Dysregulation of CTSS, along with CTSL expression, has been reported to modulate the pool of peptides presented to CD4 T‐cells *in vitro*, either by aberrant peptide cleavage or by facilitating class II loading in a different compartment, with a potentially different peptide pool.^76^ In fact, increased IL‐6‐dependent CTSS expression in Blimp‐1‐deficient DCs altered antigen processing and ultimately skewed differentiation of CD4 T‐cells into Tfh cells bearing a diverse TCR Vβ repertoire associated with autoimmunity. Adding weight to the observations in mice, patients with SLE and lupus nephritis present with increased CTSS serum levels.^80^ GM‐CSF + IL‐4‐cultured Mo‐DCs from female SLE‐risk allele carriers (rs548234) were also found to exhibit lower *Prdm1* expression, and elevated *ctss* and HLA‐DR expression at steady‐state.^78^, ^81^

Blimp‐1 also directly interferes with the expression of peptide presentation machinery by suppressing transcription of the co‐activator Class II major histocompatibility complex transactivator (CIITA), which serves as a master regulator for the expression of MHC Class II genes (reviewed in Ref. [82]). Indeed, female splenic Blimp‐1‐deficient DCs were reported to present with constitutively increased MHC Class II expression *in vivo*.^72^ The reduction in CIITA expression also occurs in human (GM‐CSF + IL‐4) Mo‐DCs and murine GM‐CSF‐cultured BMDCs in steady‐state, and upon LPS, TNFα, CD40L and IFNα stimulation, as well as infection with *Salmonella typhimurium* and Sendai virus.^53^, ^83^ Consistent with the rapid induction of *Prdm1* by multiple stimuli, as discussed above, the kinetics of Blimp‐1 expression in human Mo‐DCs inversely correlates with CIITA expression upon DC activation, consistent with its role in B‐cells during B‐cell to plasma cell differentiation.^84^, ^85^

**Figure 3:**
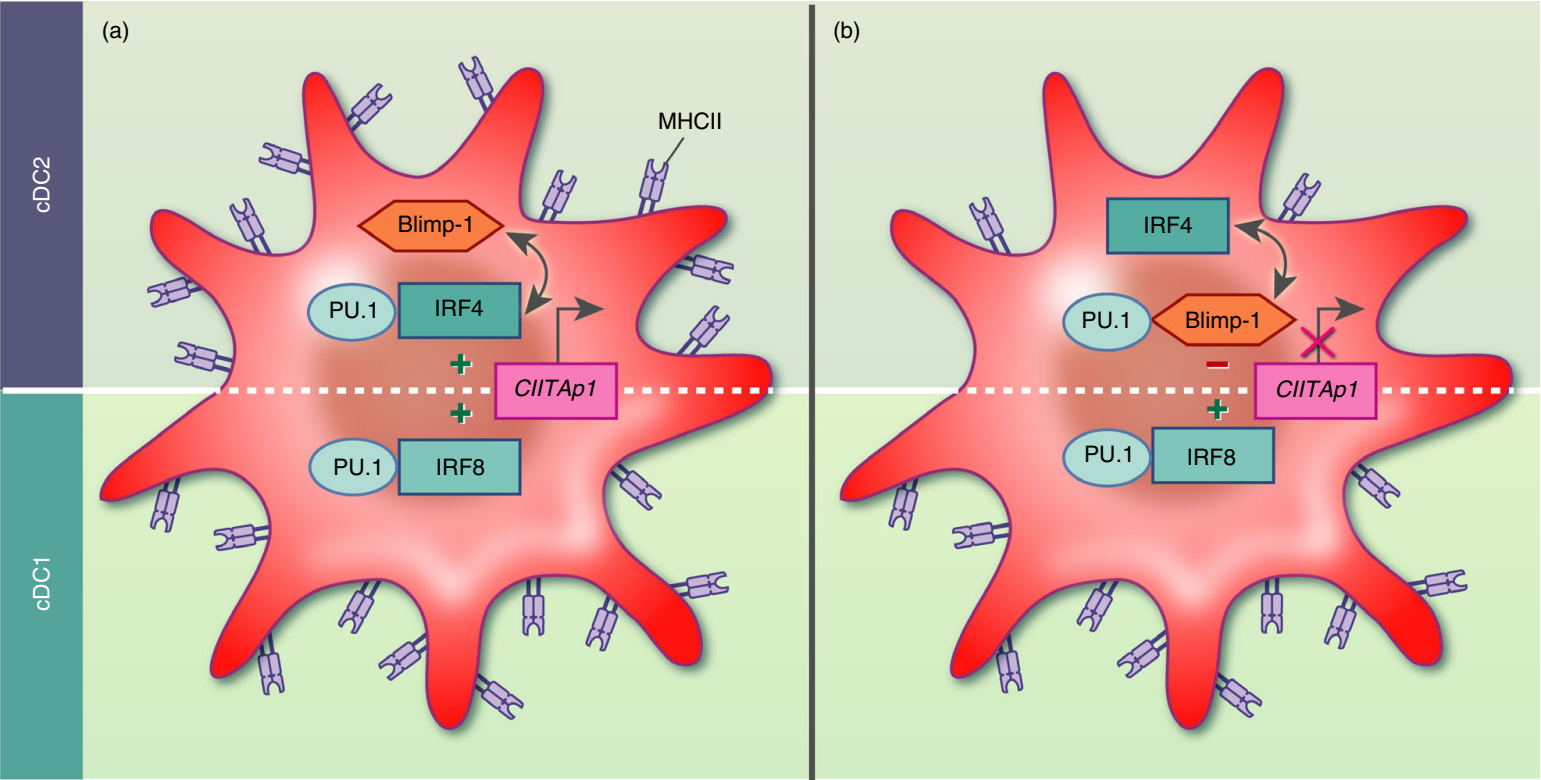
A model for B lymphocyte‐induced maturation protein‐1 (Blimp‐1)‐meditated attenuation of MHC Class II expression via silencing the MHC Class II‐transactivator CIITA in dendritic cells (DCs). (a) Interferon regulatory factor (IRF)8 and IRF4, transcription factors mutually exclusive to cDC1 and cDC2 respectively, in a complex with PU.1, facilitate promoter assembly (CIITAp1) to activate transcription of CIITA resulting in the downstream activation of MHC Class II. (b) Blimp‐1 expression in cDC2 potentially silences CIITA expression by displacing IRF4. This results in the disassembly from the promoter, followed by chromatin remodelling, effectively controlling MHC Class II expression. IRF4 can directly induce Blimp‐1 in response to maturation and/or activation signals.

CIITA expression is under the control of four independent promoters in humans (pI−pIV), and three in mice (pI, pIII and pIV). Transcription of CIITA from pI is restricted to cDCs and macrophages, while MHC Class II expression in the lymphoid lineage is primarily regulated by CIITApIII (reviewed in Ref. [86]). *In vivo* genomic footprinting analysis complemented with ChIP analysis on human LPS‐stimulated Mo‐DCs showed that Blimp‐1 silences CIITA expression by displacing an IRF8/PU.1 complex at CIITApI during DC activation (Fig. 3). Stable silencing is further reinforced epigenetically by Blimp‐1‐mediated recruitment of the chromatin‐modifying enzymes G9α and HDAC2 to the promoter, resulting in a repressed chromatin state.^84^ Although the Ets‐IRF composite element of CIITApI is able to recruit both IRF8 and IRF4 in a complex with PU.1,^87^ Smith *et al*.^84^ reported dominance of IRF8 in the contribution to CIITA activation. Concomitantly, B‐cells utilize IRF4 and PU.1 (among others) for CIITApIII promoter activation.^88^, ^89^ IRF4 or IRF8 reconstitution of GM‐CSF + IL‐4‐cultured BMDCs from IRF4‐deficient DC progenitors could, however, similarly recover CIITA expression, in line with comparable expression levels of MHC Class II in both IRF8‐dependent cDC1 and IRF4‐dependent cDC2 in general. Because ChIP‐seq analysis of LPS‐treated GM‐CSF‐cultured BMDCs revealed that IRF4 induces *Prdm1*, transcription factors exclusive to the cDC2 subset, this argues for a more complicated incoherent feed‐forward loop in transcriptional regulation of antigen presentation by cDC2, specifically.^50^ Of note, IRF4 also negatively regulates cystatin C (CST3), which in turn inhibits the activity of CTSS,^50^, ^90^ suggesting an additional overlap of transcriptional targets involved in antigen presentation by IRF4 and Blimp‐1 (Fig. 2). Together, these data suggest that Blimp‐1 and IRF4 are part of a complicated network downstream of PRR engagement in the modulation of the MHC Class II antigen presentation pathway, with significant relevance for the regulation of the innate‐adaptive immune interface.

## Reported Blimp‐1‐associated polymorphisms linked to mononuclear phagocyte function

Blimp‐1 has been identified as a gene contributing to IBD pathogenesis by an extensive meta‐analysis of GWAS studies,^91^ and an exome sequencing study identified variants of Blimp‐1 single nucleotide polymorphisms (SNPs) that are associated with Crohn’s disease. Reduced *PRDM1* expression in ileal biopsy specimens and peripheral blood mononuclear cells correlated with the Crohn’s disease GWAS‐associated lead risk SNP rs7746082 among the 10 identified SNPs within the *PRDM1* region.^92^ Investigations of Blimp‐1 expression in this study were narrowed to the lymphocyte lineage and, indeed, T‐cell dysregulation is associated with colitis in many murine models (reviewed in Refs [34, 93]). However, GWAS enriched for cell‐type expression specificity of genes in IBD risk loci highlighted the strongest enrichment in DCs, suggesting that DCs are a key component of IBD pathogenesis.^94^

The SNPs rs548234 (Han Chinese)^95^ and rs6568431 (European)^96^ predispose females to the development of SLE, and are both located in the intergenic region between *PRDM1* and *ATG5*. Further analysis of the Han Chinese SNP revealed that DCs, but not B‐cells, show lower Blimp‐1 expression in individuals carrying the risk allele,^81^ while *ATG5* expression was unchanged. As expected, lower Blimp‐1 expression further correlated with heightened let‐7c miRNA and HLA‐DR expression. Interestingly, the SNP induces binding of the transcriptional repressor KLF4 (kruppel‐like factor 4), which is expressed at high levels in DCs, providing a mechanistic explanation for why alterations in Blimp‐1 levels are specific to DCs, and cementing the finding that dysregulation of DCs, caused by low Blimp‐1 expression, can lead to SLE.^81^

## Outlook

Taken together, a picture emerges in which Blimp‐1 fulfills critical roles in the maintenance of immune homeostasis by integrating environmental triggers and imprinting context‐specific function of MNPs. Despite increasing recognition of the potential of Blimp‐1 as a powerful rheostat of immune activation, little is known about its *in vivo* regulation and its defined targets in the MNP system. This is mostly due to both the intrinsic heterogeneity of MNP subsets and the highly contextual expression patterns of Blimp‐1. Novel technologies including single‐cell RNA sequencing across tissues and immunological states will continue to pave the way for innovative approaches to modulate immune activation by harnessing Blimp‐1.

## Conflict of interest

The authors declare no conflict of interest.

## Data Availability

No new data were created for this manuscript.
